# A Novel Marmoset (*Callithrix jacchus*) Model of Human Inhalational Q Fever

**DOI:** 10.3389/fcimb.2020.621635

**Published:** 2021-01-28

**Authors:** Michelle Nelson, Francisco J. Salguero, Laura Hunter, Timothy P. Atkins

**Affiliations:** ^1^ CBR Division, Defence Science and Technology Laboratory (Dstl), Salisbury, United Kingdom; ^2^ Public Health England, Salisbury, United Kingdom

**Keywords:** nonhuman primate, infectious disease, histology, innate immunology, *Coxiella burnetii*

## Abstract

Common marmosets (*Callithrix jacchus)* were shown to be susceptible to inhalational infection with *Coxiella burnetii*, in a dose-dependent manner, producing a disease similar to human Q fever, characterized by a resolving febrile response. Illness was also associated with weight loss, liver enzyme dysfunction, characteristic cellular activation, circulating INF-*γ* and bacteraemia. Viable *C. burnetii* was recovered from various tissues during disease and from 75% of the animal’s lungs on 28 days post challenge, when there were no overt clinical features of disease but there was histological evidence of macrophage and lymphocyte infiltration into the lung resulting in granulomatous alveolitis. Taken together, these features of disease progression, physiology and bacterial spread appear to be consistent with human disease and therefore the common marmoset can be considered as a suitable model for studies on the pathogenesis or the development of medical counter measures of inhalational Q fever.

## Introduction

*Coxiella burnetii* is a Gram negative intracellular bacterium and the causative agent of the zoonotic disease Q fever. The primary reservoir of disease is farm animals, such as cattle, sheep and goats (McQuiston and Childs, 2002). Infection in humans usually occurs *via* the inhalation of aerosolized products from infected animals, such as urine, faeces or birth fluids. Q fever has an almost ubiquitous worldwide distribution, with the largest described outbreak of Q fever occurring in the Netherlands between 2007–2010. This outbreak resulted in >4,000 human cases and >50,000 goats culled in order to control spread of the disease, resulting in an estimated economic loss of over 300 million euros (Whelan et al., 2011; Dijkstra et al., 2012; van Asseldonk et al., 2013). In addition, there are several reports of outbreaks amongst military personnel serving in Iraq and Afghanistan (Moodie et al., 2008; Faix et al., 2008; Bailey et al., 2011).

Recommended treatment for acute Q fever is 100 mg of doxycycline for 14 days (Anderson et al., 2013). Combination therapy with hydroxychloroquine reduces the likelihood of developing endocarditis in acute patients with predisposing factors such as valvulopathy (Maurin and Raoult, 1999). However, additional novel medical countermeasures are required because of poor tolerance to doxycycline and inadequate treatment of acute Q fever has been identified as a potential risk factor for developing chronic Q fever (Kampschreur et al., 2012). In addition, there is an emergence of doxycycline-resistant strains of *C. burnetii* which has the potential to impact treatment options in the future (Eldin et al., 2017).

Mice and guinea pigs have been routinely used to model Q fever, to characterize pathogenesis of disease and evaluate medical countermeasures (Bewley, 2013). Pyrexia is observed in guinea pigs challenged with *C. burnetii* by both the intra-peritoneal and inhalational route which is lethal at high challenge doses (Moos and Hackstadt, 1987; Russell-Lodrigue et al., 2006). Mice are relatively resistant to *C. burnetii*, however male A/Jola (A/J) mice are susceptible (Scott et al., 1978; Scott et al., 1987). Disease can be characterized using five parameters: weight loss, clinical signs, organ weight, bacterial burden, and histopathology (Hartley et al., 2019). A/J mice have been used to assess efficacy of antibiotics (Norville et al., 2014). Nonhuman primate (NHP) models including the cynomolgus and rhesus macaque have also been used to model Q fever (Gonder et al., 1979; Kishimoto et al., 1981; Waag et al., 1999; Waag et al., 2002). In these studies, animals exhibited a febrile response, radiographic changes in the lungs and resolving bacteraemia following aerosol challenge (Waag et al., 1999). In addition, the cynomolgus macaque model was successfully used to demonstrate protective efficacy using a whole killed cell vaccine formulation (Waag et al., 2002).

However, there are few recent studies reported that fully characterize the disease in the context of the human syndrome. This paper reports the development of a model of inhalational Q fever using an alternative nonhuman primate, the common marmoset *(Callithrix jacchus)*. This model has been characterized and has potential utility to assess novel medical countermeasures against Q fever for human use.

## Materials and Methods

### Animals

Healthy, sexually mature common marmosets (*C. jacchus*) were obtained from the Dstl Porton Down breeding colony and housed in vasectomized male and female pairs. The mean age and weight of the animals used 1.9 ± 0.6 years and 410 ± 36g at the time of challenge. All animals were allowed free access to food and water as well as environmental enrichment. All animals were surgically implanted intraperitoneally with a Remo 200 device (EMMS, Bordon, Hampshire, UK) under general anaesthesia (Ketamine/Domitor/Isofluorane) to record core body temperature (Tc). Data were transmitted from the devices at 30 second intervals to locally placed antennas and relayed to receivers. Data were analyzed using the eDacq software to provide real-time and recordable Tc (EMMS, Bordon, Hampshire, UK). The animal studies were carried out in accordance with the UK Animals (Scientific Procedures) Act of 1986 and the Codes of Practice for the Housing and Care of Animals used in Scientific Procedures 1989. Following challenge with *C. burnetii*, animals were handled under animal containment level 3 (CL3) conditions, within a half-suit isolator compliant with British Standard BS5726.

### Bacterial Strain and Culture

*C. burnetii* Nine Mile Phase I (RSA493) was cultured axenically in ACCM-2 (Omsland et al., 2011), in a vented conical flask within a sealed container at 37°C, shaking at 75 rpm for 6 days, with a GENbox microaer generator (bioMérieux, France) to displace oxygen. Prior to challenge, bacteria were pelleted *via* centrifugation and stored at -80°C in phosphate buffered saline (PBS) until required.

For enumeration of bacteria, viable counts were performed using ACCM-2 solid media (for challenge counts, and tissue burden), the FilmArray system (for DNA detection in blood samples) and real-time quantitative PCR (qPCR; for challenge counts only). Viable counts were performed by ten-fold serial dilution and plating multiple 100 µl aliquots onto ACCM-2 solid media and incubated statically for 14 days at 37°C with the atmosphere adjusted to 5% CO_2_ and 2.5% O_2_ (Galaxy 170 R incubator, New Brunswick Scientific, UK). For quantification of total bacterial DNA, *C. burnetii* was enumerated using qPCR targeting the *com1* gene (Hartley et al., 2019). Primers and probe were purchased (ATDBio Ltd., Southampton, UK). Quantification of genome equivalents (GE) was conducted by qPCR using a viable culture of known CFU to develop a standard curve (Hartley et al., 2019).

The Bio Threat panel on the FilmArray system (BioFire Diagnostics Inc., Salt Lake City, USA) was used to test for bacterial DNA in blood samples and used according to the manufacturer’s instructions with the exception that 100 µl rather than 200 µl of blood was tested. The system detects the multi target gene IS1111.

All manipulations of *C. burnetii* were carried out in a Class III microbiological safety cabinet, complying with British Standard EN12469:2000.

### Aerosol Challenge

Prior to challenge animals were anaesthetized with 10 mg/kg of ketamine hydrochloride *via* the intramuscular route. Briefly, an aerosol was generated using a 6-jet Collison nebulizer containing a 10 ml suspension of the appropriate concentration of *C. burnetii* using a contained Henderson apparatus controlled by the AeroMP (Aerosol Management Platform) system (Biaera Technologies L.L.C, MD USA) Animals were placed within a plethysmography tube and attached to the exposure unit as previously described (Nelson et al., 2009). Pairs of animals were exposed to the aerosol for 10 min *via* a head-only exposure chamber, with samples impinged from the chamber using an AGI-30 (Ace Glass Inc., USA) containing PBS at 12 L/min. The accumulated volume of air breathed by each animal was determined real-time using eDacq software (Version 1.8.4b). The dose each animal received was calculated as follows:

$$\begin{array}{l} \text{Aerosol Concentration}\;(\text{cfu/L of air})\\ =\frac{\text{Impinger count (cfu}/\text{mL)}\times \text{ Impinger volume (ml)}\;\;\;\;\;\;\;\;\;\;\;\;\;\;\;\;\;\;}{\text{Impinger flow rate (L}/\text{minute)}\times \text{ Impinger time }(\text{minutes})} \end{array}$$

$$\begin{array}{l} Dose\;received\;(cfu)\\ =\;\text{ Aerosol concentration (cfu }/\text{L of air) }\times \text{ total accumulated volume (L)} \end{array}$$

### Post-Mortem Analysis

Animals were euthanized at 21 days (dose-ranging studies) or scheduled time-points post-challenge (pathogenesis study) and organs were aseptically removed, weighed and examined for gross pathological changes. For bacteriology, weighed sections were homogenized into 1 ml PBS and enumeration of bacterial load determined as described above. In addition, liver, spleen, kidney, lungs, genitals, and lymph nodes were placed in 10% neutral buffered formalin for histological analysis. In addition, blood was collected *via* a post-mortem cardiac puncture into sodium citrate (bacteriological and immunological analysis), lithium heparin (clinical chemistry analysis) and EDTA tubes (haematology analysis). Concentrations of albumin, alanine aminotransferase (ALT), aspartate aminotransferase (AST), alkaline phosphatase (ALKP), amylase, total bilirubin, blood urea nitrogen, calcium, phosphate, total protein, creatine, and glucose levels were measured using a Catalyst Dx (Idexx Laboratories, Inc.). Haematology levels (red blood cells, white blood cells, haemtocrit, platelets, neutrophils, monocytes, eosinophils and basophils) were measured using a Procyte Dx (Idexx Laboratories, Inc.).

### Histopathological Analysis

Sections of tissue from all animals were fixed in 10% neutral buffered formalin, processed to paraffin wax and 3–5 µm thick sections cut and stained with haematoxylin and eosin (HE). Tissues were examined by light microscopy and evaluated in a blinded manner. Slides from age-matched unchallenged animals were evaluated to establish the nature of background, incidental lesions. Imagic IMS software was used to capture and store digital images.

### Cell Type Determination

Blood samples or single cell suspensions of lung and spleens were used for immunological analysis. Red blood cells were lysed using RBC lysis buffer (BD Biosciences, CA, USA) and the remaining leukocytes were stained using three sets of five to seven antibody bound fluorescent stains to identify cell phenotypes and activation status by flow cytometry. Samples were inactivated using 4% PFA for 40 h at 4°C prior to Flow cytometry using a BD FACS Canto II cytometer and BD FACS Diva software. Whole cells were detected by nuclear staining allowing the area of interest to be defined by forward and side scatter. Forward and side scatter were also used to gate areas for detection of lymphocytes (T and B cells), natural killer cells (NK), macrophages (M0), and neutrophils. Fluorescent bound mouse anti-human antibodies included for lymphocytes CD3, CD8, CD56, CD69, CD20, and CD16, and for monocyte/macrophages and neutrophils CD163, CD14, CD11c, CD80, HLA-DR, CD40, CD64, and CD54 (BD Biosciences USA, and BioLegend USA).

### Cytokine Analysis

Plasma and supernatant fluid collected from homogenised lung and spleen tissue were stored at -80°C. Samples were thawed and analyzed using human antibodies from BD™ Cytometric Bead Array (CBA) Flex set [IL-6, IL-1β, MCP-1(CCL2), MIP-1β (CCL4), and RANTES (CCL5)]. In order to identify IFN*γ* and TNFα, custom made kits were specifically manufactured by BBI Detection Ltd UK using marmoset specific antibodies produced by U-Cytech biosciences, Netherlands and Mabtech AB, Sweden. Processing followed BD flex set instructions. Samples were inactivated using 4% PFA for 40 h at 4°C prior to Flow cytometry.

### Serological Analysis

*C. burnetii* Phase I and II IgG antibody detection ELISA kits (NovaTec, GmbH, Germany) were used to test the antibody response of plasma from the marmosets. The assay was performed in accordance with to the manufacturer’s instructions with the exception of the detection antibodies. An alternative human IgG detection antibody (The binding site UK) was used at 1:1,000. Samples were considered positive if the optical density was more than 2 standard deviations above the mean value of pre-exposure marmoset sera.

### Re-Stimulation Assay

Single cells suspensions from spleen homogenates of an estimated 1x10^6^ cells/ml were decanted into L-15 (plus glutamax and 10% foetal calf serum plus penicillin and streptomycin, Gibco, UK) in 96 well plates and exposed to media only (control), ConA 2.5 µg/ml (Sigma UK), heat killed Phase I and heat killed Phase II, both at 1x10^7^ *C. burnetii*/ml, and incubated at 37°C for 24 h. The supernatant was removed and stored at -80°C until analysis for levels of IFN*γ*, (performed as for cytokine analysis).

### Statistical Analysis

Haematological and clinical chemistry parameters in the blood were compared for each animal pre-challenge and with blood collected at the time of post mortem using one-way ANOVA analysis (with some data transformed by Log_10_ to ensure normal distribution). Bacterial load data was transformed by Log_10_ and analyzed by one-way ANOVA. Time and duration of fever was analyzed by Pearsons’ Correlation. Any statistical significance is highlighted in figures using an asterisk.

## Results

### Dose Characterization

In order to evaluate the susceptibility of marmosets to *C. burnetii* and monitor the disease progression, two studies were performed. Initially, a dose-ranging study assessed the virulence of *C. burnetii* where animals were challenged with between 2 and 8.5 x 10^5^ GE of bacteria *via* inhalational route and observed for 21 days post-challenge (p.c.; **Table 1**; Hartley et al., 2019). Quantification of GE was achieved by comparison to a known viable culture. In the second study, marmosets were challenged with a mean dose of 1.2 x 10^4^ GE of *C. burnetii*. Two pairs of animals were euthanized at predetermined time points (3, 7, 14 and 28 days p.c.) to assess disease pathogenesis.

**Table 1:** Summary of challenge dose, fever and weight loss for animal challenged with *C. burnettii* by the inhalational route.

Study ID	Inhaled dose (GE)	Time of euthanasia (days post-challenge)	Time to onset of fever (days)	Length of fever (days)	Max weight loss (% total body weight)
Dose-ranging study	2	21	NF	NF	WG
	6	21	NF	NF	WG
	8.7 x 10^2^	21	4.25	2.5	0.5
	5.1 x 10^3^	21	5.25	7.75	4
	8.0 x 10^3^	21	5.25	6.5	6.5
	1.8 x 10^4^	21	5.25	6.5	2
	4.6 x 10^4^	21	5.25	2.5	WG
	7.2 x 10^4^	21	5.25	6.5	6.5
	4.3 x 10^5^	21	4.25	7.5	6
	5.6 x 10^5^	21	4.25	7.5	2.5
	6.4 x 10^5^	21	4.25	7.5	0
	8.2 x 10^5^	21	4.25	7.5	8.5
Pathogenesis study	1.7 x 10^3^	3	^1^NF	^1^NF	2.5
	6.6 x 10^3^	3	^1^NF	^1^NF	WG
	6.9 x 10^3^	3	^1^NF	^1^NF	WG
	8.5 x 10^3^	3	^1^NF	^1^NF	2.5
	1.4 x 10^4^	7	5.5	^2^ND	2
	1.7 x 10^4^	7	5.75	^2^ND	WG
	1.9 x 10^4^	7	6	^2^ND	WG
	2.2 x 10^4^	7	5.75	^2^ND	ND
	8.2 x 10^3^	14	5.5	7.5	6.5
	9.6 x 10^3^	14	5.75	4.25	0.5
	1.3 x 10^4^	14	5.5	7.5	11
	1.5 x 10^4^	14	5.5	6.5	0.5
	3.6 x 10^3^	28	5.5	11	2
	1.3 x 10^4^	28	6.5	8	WG
	1.7 x 10^4^	28	6.25	6.75	WG
	1.9 x 10^4^	28	5.25	9.75	8
Mean	1.2 x 10^4^ ± 1.5 x 10^3^	n/a	5.7 ± 0.1	7.7 ± 0.7	3.9 ± 1.2

NF is no fever observed; WG is weight gain observed; ND is not determined.

^1^Animals culled prior to the onset of fever.

^2^Animals culled during the febrile period.

GE determined by comparison to known viable standard (Hartley et al., 2019).

**Figure 1:**
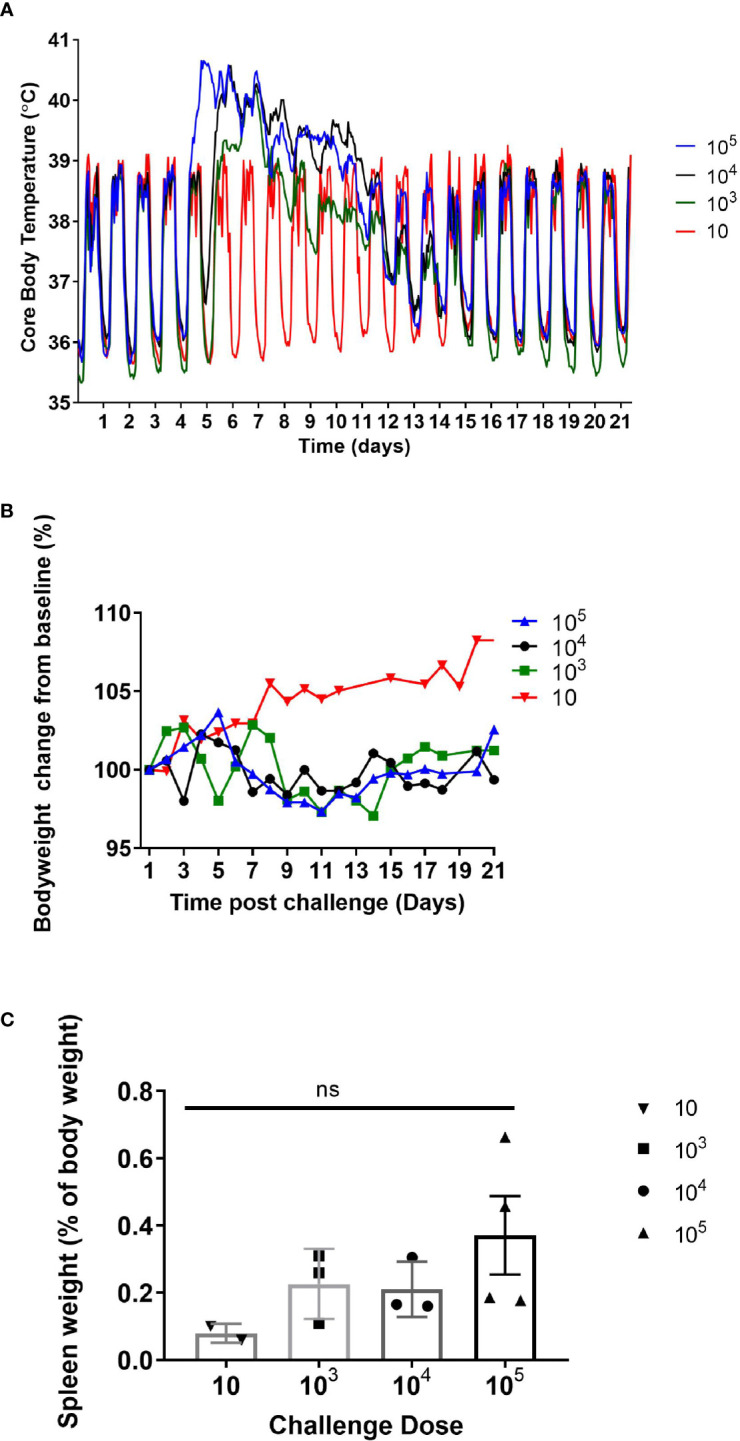
**(A)** Mean core body temperature, **(B)** body weight profile, and **(C)** spleen weight (at day 21 post challenge) from marmosets challenged with 10-10^5^ GE of *C*. *burnetii* by the inhalational route. 10 GE: n=2 (range: 2-6 GE); 10^3^ GE: n=3 (range: 8.7 x 10^2^ – 8.0 x 10^3^ GE); 10^4^ GE: n=3 (range: 1.8 – 7.2 x 10^4^ GE); 10^5^ GE: n= 4 (range: 4.3 – 8.2 x 10^5^ GE). Error bars represent the mean and SEM. NS = not significant as determined by one-way ANOVA.

There was an absence of overt clinical signs in all the animals during the monitoring period (21 or 28 days p.c.). However, 50% of the animals showed subtle changes in behaviour starting at day 6 or 7 p.c. The animals were subdued, had reduced activity, somnolent with unkempt fur on initial observation, however, following stimulation their behaviour returned to normal. This continued in all animals until at least day 8, with two animals remaining subdued for a further 2 days. All animals challenged with *C. burnetii* exhibited a febrile response, except the two animals that received a low dose of *C. burnetii* (less than 10 cfu; **Figure 1A**). These two animals had a normal temperature and diurnal rhythm for the duration of the study. All remaining animals became febrile between 4.25 to 6.5 days p.c. (mean time of 5.3 ± 0.7 days). Onset of fever (defined as greater than 40°C) was statistically correlated to the dose the animals received (Pearsons’ Correlation R^2^ = 0.60, P = 0.0081). However the duration of fever varied from 2.5 to 9.75 days (mean of 6.9 ± 2.1 days) and was not associated with the dose the animals received. Typically, the body weight of all animals, except those challenged with less than 10 cfu of bacteria, declined between day 5 to 15 p.c. (**Figure 1B**). Animals lost between 2 and 11% (mean of 4.0 ± 3.2%) of their initial body weight, compared to an 8% gain in weight observed in the low dose animals. In addition, the weight of the spleen from infected marmosets at 21 days p.c. increased with challenge dose (**Figure 1C**).

### Bacteriology and Physiological Response Following *C. burnetii* Challenge

**Figure 2:**
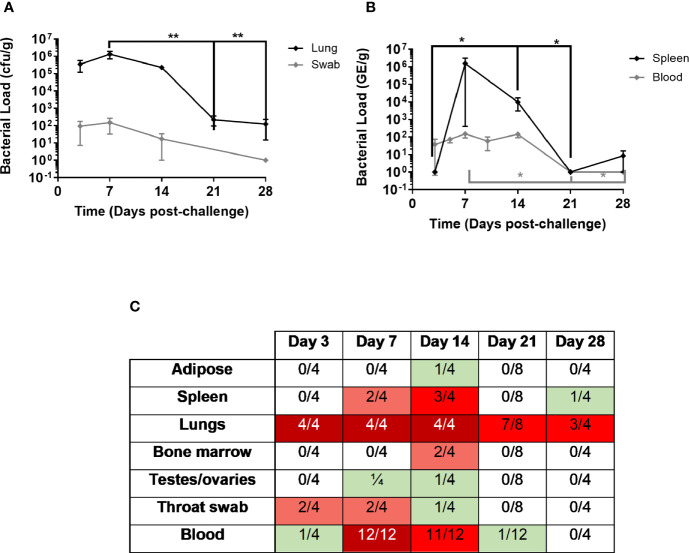
Bacterial load in marmoset tissues over time following inhalational challenge with a mean dose of 1.16 x 10^5^ GE of *C*. *burnetii* (range 8x10^3^–5x10^5^). At least four animals were euthanized at each time point. **(A)** Lung bacteriology (black diamond cfu/g) and throat swab (grey diamond GE/swab) **(B)** bacteremia (grey diamond cfu/ml) and spleen bacteriology (black diamond cfu/g) **(C)** Heat map to show number of animals with colonized adipose, spleen, lungs, bone marrow, testes/ovaries, throat swab, and blood. Data is median and interquartile range, significance determined by ANOVA; *p<0.05, and **p<0.01.

In the pathogenesis study, levels of culturable bacteria in lung, spleen, adipose tissue, femurs, testes/ovaries and throat swabs were assessed at the time of post mortem (Day 3, 7, 14, 21, & 28; **Figure 2**). Bacteraemia was assessed from blood collected at day 7 or day 8 and day 14 p.c. (study 1 and 2) and day 5 & 10 p.c. (study 2), and at the time of post mortem. At day 3 p.c., a mean concentration of 3.5 x 10^5^ cfu/g *C. burnetii* were cultured from the lungs of the animals euthanised at this time. One animal also had 1.5 x 10^2^ cfu/ml of bacteria cultured from the blood. By day 5 p.c., low levels of bacteraemia was observed in all animals assessed (9.9 x 10^1^; **Figure 2B**). Bacteria continued to proliferate in the lungs, with a mean concentration of 2.9 x 10^6^ cfu/g *C. burnetii* cultured from all animals euthanised at day 7 p.c (**Figure 2B**). Bacteria had also disseminated to other tissues at this time, with 2 out 4 animals having up to 1.4 x 10^4^ cfu/g in the spleen and one animal having 4.8 x 10^2^ cfu/g in the testes (**Figure 2C**). Positive throat swabs were detected in 2 out 4 of the animals and all animals had bacteraemia. In the dose-ranging study, bacteria were detected in the blood at day 7 or 8 p.c. in all animals that received a dose of > 1 x 10^2^ (83% of animals); above a challenge of 1 x 10^3^ there was no relationship between challenge dose and blood viable count. Levels of *C. burnetii* in the lungs decreased by day 14 with a mean concentration of 2.3 x 10^5^ cfu/g, with 3 animals also having bacteraemia and bacteria detected in the spleens of two animals (up to 3 x 10^4^ cfu/g), the testes of one animal (5.4 x 10^3^ cfu/g) and the adipose of one animal (1.4 x 10^3^ cfu/g). As the disease resolved, on day 21 a low level of bacteria was cultured from the blood of one animal (1/8), however 7/8 of the animals had culturable bacteria recovered from their lungs. *C. burnetii* was also detected in the lungs of 3/4 animals at day 28 and one animal had bacteria in the spleen (**Figure 2C**).

PCR was performed on all of the blood samples using the Biothreat panel in the FilmArray system. All blood samples were PCR negative on day 3 (despite one being culture positive), but thereafter all samples including the 4 from day 28 were PCR positive (data not shown).

**Figure 3:**
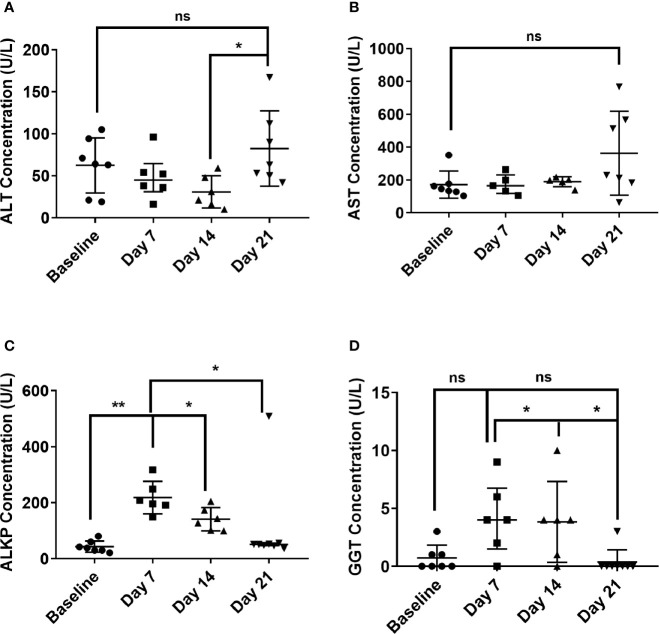
Temporal liver enzymes changes in marmoset blood at day 7, 14 and 21 following inhalational challenge with *C*. *burnetii.* **(A)** alanine transaminase (ALT), **(B)** Aspartate aminotransferase (AST), **(C)** alkaline phosphatase (ALKP), **(D)** gamma-glutamyl transferase (GGT). Data is median and interquartile range, significance determined by ANOVA; indicated by asterisks * p<0.05, and **p<0.01.

Haematological and clinical chemistry parameters from all animals were compared from pre-challenge blood and blood collected at the time of post mortem. There was no significant difference in the haematological parameters pre and post-challenge. There were some minor differences in the clinical chemistry parameters, most notably a general increase in the glucose and lactate dehydrogenase levels at post mortem. Liver enzyme dysfunction was also noted from post-challenge blood collections at day 7 or 8 and day 14, and at the time of post-mortem (day 21; **Figure 3**). Levels of two enzymes, aspartate aminotransferase (AST) and alanine transaminase (ALT), were only abnormally raised at day 21 p.c. (**Figures 3A, B**). However, levels of gamma-glutamyl transferase (GGT) and alkaline phosphatase (ALKP) increased at day 7 or 8 p.c. and remained elevated at day 14 before returning to normal levels by Day 21 (**Figures 3C, D**).

### Immunological Response to Inhalational *C. burnetii*

**Figure 4:**
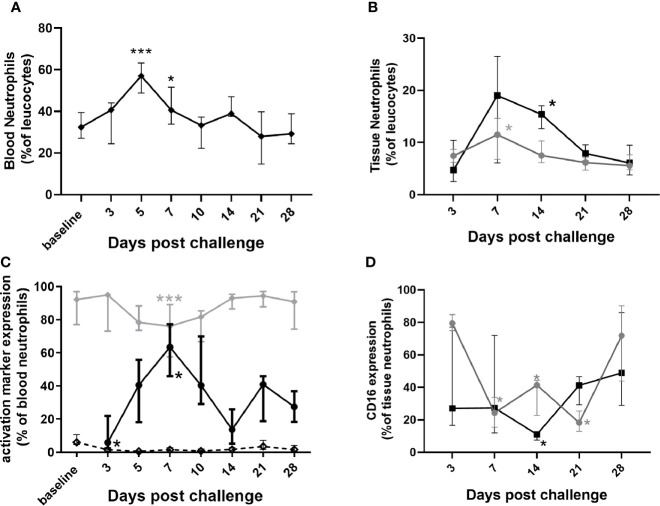
Changes in neutrophil populations in marmoset tissues at following inhalational challenge with *C*. *burnetii*. **(A)** blood **(B)** lung (grey circle) and spleen (black square) homogenates: **(C)** changes in activation/maturity marker expression CD16+ (dotted line), HLA-DR+ (gray diamond), and CD64+ (black circle)] on neutrophils in blood and **(D)** CD16+ expression on lung (gray circle) and spleen (black square) neutrophils. Data is median and interquartile range, significance from baseline or Day 3 by ANOVA; *p<0.05, and ***p<0.001.

In general, neutrophil changes typically associated with mild infection were observed in marmosets. There was an increase in the percentage of circulating blood neutrophils, apparent as early as day 3 p.c., significantly higher at day 5 and remaining elevated on day 7 (**Figure 4A**). There was also an increase in the percentage of neutrophils in the lung by day 7 p.c. and in the spleen by day 14 p.c., presumably following the spread of bacteria (**Figure 4B**). There was also a change in activation markers expressed by the neutrophils, for example CD16^+^ (a marker of neutrophil maturity) was significantly reduced on circulating neutrophils from the day 3 p.c. (**Figure 4C**) and in the lungs at day 7 and in the spleen day 14 (**Figure 4D**). Other markers of neutrophil health were also affected, with CD64^+^ (a sepsis marker in humans) significantly increased on circulating neutrophils by day 7 p.c. and reduced by day 14. The reverse profile was seen with neutrophil HLA-DR^+^ expression (**Figure 4C**) both in line with the level of bacteraemia.

**Figure 5:**
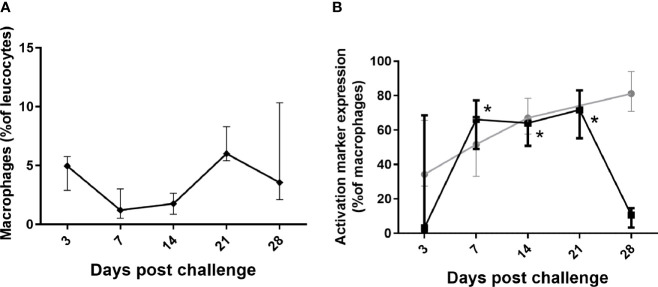
Changes in lung macrophages in marmosets following inhalational challenge with *C*. *burnetii*.** (A)** macrophages as a proportion of leucocytes and **(B)** change in expression markers, CD16 (gray circle) and CD40 (black square) on macrophages. Data is median and interquartile range, significance from Day 3 by ANOVA; indicated by asterisks; *p<0.05.

Changes were observed with the monocytes/macrophages that were consistent with controlling and clearing a bacterial infection (**Figure 5**). There was a significant increase in CD40^+^ expression (classical activation associated with increased phagocytosis and bacterial killing) on lung macrophages, lasting from day 7 until day 28, in line with bacterial clearance; (**Figure 5B**), and also in the blood and spleen significant for day 10–14 p.c. (data not shown). There was a steady (but not significant) increase in expression of CD16^+^ (associated with alternative activation, tissue repair and recovery) on both lung (**Figure 5B**) and blood macrophages from day 7 p.c. onwards.

**Figure 6:**
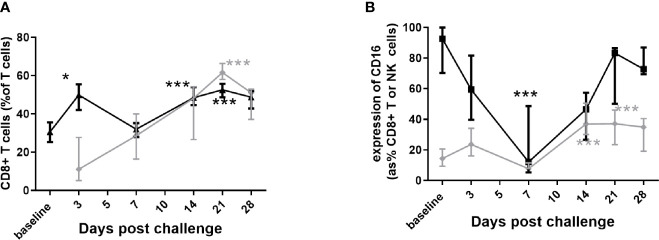
Changes in the lymphocyte populations in marmoset tissues following inhalational challenge with (*C*) *burnetii*. **(A)** changes in the CD8+ percentage of T cells in blood (black triangle) and lung (grey diamond), and **(B)** changes in CD16+ expression on CD8+ T cells (grey diamond) and on NK cells (CD3- CD56+ black square) in blood. Data is median and interquartile range, significance from baseline or Day 3 by ANOVA; * p<0.05, and ***p<0.001.

Involvement of lymphocytes was observed in both the lungs and the blood, but not in the spleen. There was a significant increase in CD8^+^ T cells in the blood (**Figure 6A**) and they were significantly more activated due to expression of CD16^+^ (**Figure 6B**). There was noticeable increase in the NK cells in the blood, but with a significant decrease in CD16^+^ expression (human marker of a component NK cell) during the disease (**Figure 6B**). Shedding of CD16^+^ can further activate the cytotoxic function of NK cells. Both CD8^+^ T and NK cells increased in the lung, with increased CD16^+^ expression, although not achieving significance (data not shown). There was a transient decrease in cytotoxic CD8^+^ T cells in the blood while the animals were bacteremic.

**Figure 7:**
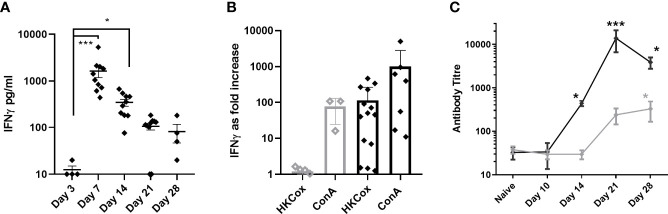
Immune response in marmosets following an inhalational challenge with (*C*) *burnetii.* **(A)** Circulating levels of IFN*γ*. Significance by ANOVA indicated by asterisks ***p<0.001 and *p<0.05 **(B)** fold increase in IFN*γ* production (above baseline) following stimulation of Day 21 or 28 (black diamond) or Day 3 (gray diamond) viable spleen cells to heat-killed (HK) *Coxiella burnet*ii or ConA (control), **(C)** antibody response to Phase I (gray diamond) and Phase II (black diamond) antigens. Data is presented as the mean and standard error.

Only two cytokines from those assessed, TNF-α and IFN-*γ* showed any relationship to clinical signs and bacteriology. IFN-*γ* was significantly elevated in the blood at day 7 p.c., declining by day 14 p.c. and back to baseline by day 21 p.c (**Figure 7A**). IFN-*γ* was also elevated in lung samples on day 7 p.c. in 75% of animals and all of the animals on day 14 p.c., returning to baseline by day 21 (data not shown). TNF-α was raised in the same lung samples, but also detected above baseline in all samples on day 21 p.c., and in 50% on day 28 (data not shown). TNF-α was not detected in the blood, and neither IFN-*γ* nor TNF-α were above baseline in the spleen samples.

The concentration of IFN-*γ* in the supernatants from a spleen re-call assay was used to determine a positive cell-mediated response; 6/7 animals (day 21 or 28 p.c) had a recall response to heat killed antigen that was 5 fold greater than background compared to no response in animals tested from day 3 p.c. (**Figure 7B**). Serological levels of IgG against the Phase II *C. burnetii* were significantly elevated starting at day 14 p.c. and peaking at day 21 p.c., coinciding with the control of bacteria in the blood. Phase I IgG was detectable from day 21 p.c., increasing slowly (**Figure 7C**).

### Histopathological Analysis

**Figure 8:**
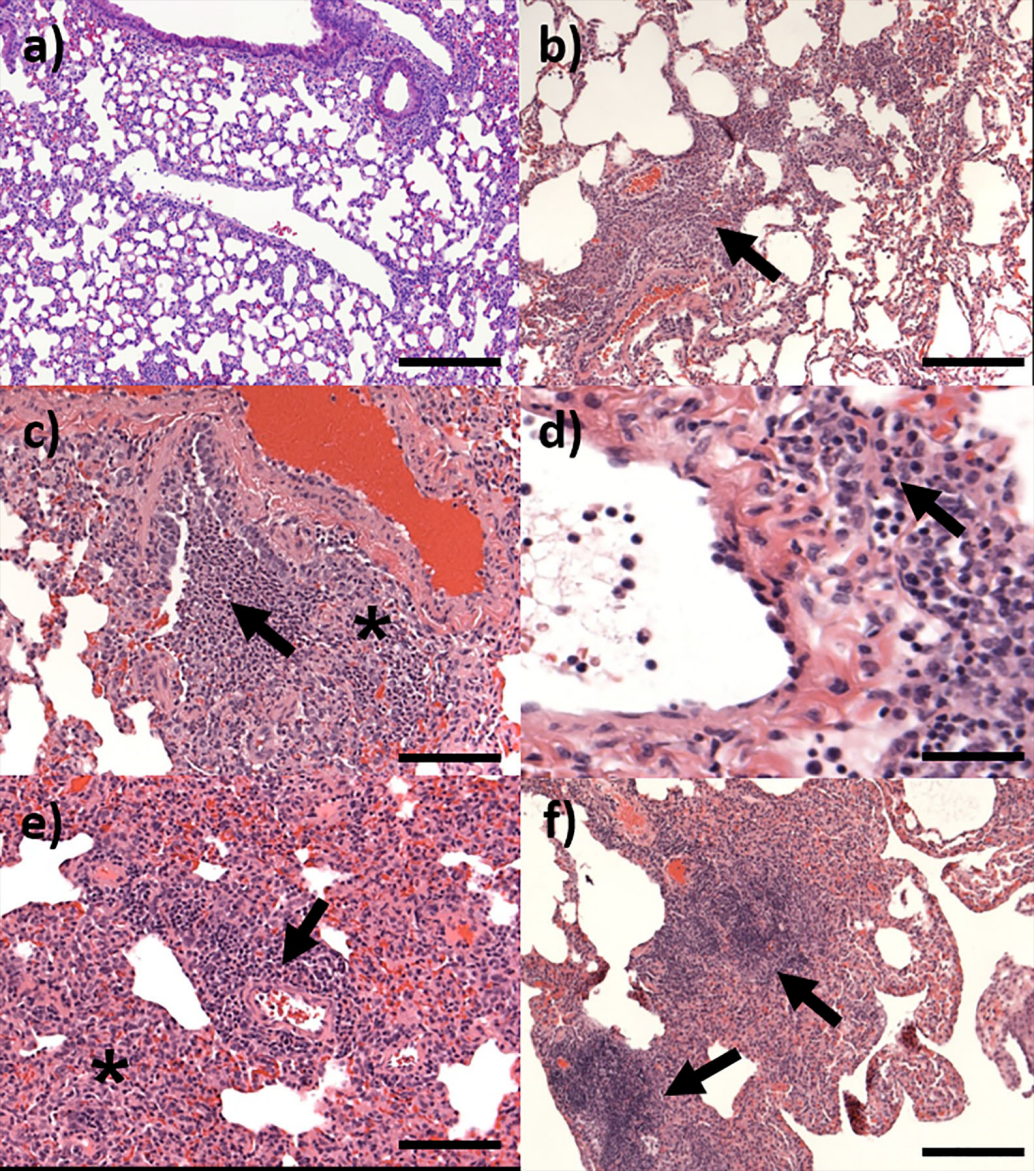
Representative H&E stained lung sections from marmosets challenged with *C*. *burnetii*. **(A)** Day 3 post-challenge showing no changes, bar =200 μm; **(B)** Day 7 post-challenge showing pyogranulomatous alveolitis (arrow), bar =200 μm; **(C)** Day 7 post-challenge showing focal bronchiolitis with infiltrating cells in the walls (*) and lumen (arrow), bar =100 μm; **(D)** Day 14 post-challenge showing perivascular cuffing (arrow) of an artery, bar=50 μm; **(E)** Day 21 post-challenge showing pyogranulomatous alveolitis (*) and lymphocytic, perivascular cuffing (arrow), bar=100 μm; **(F)** Day 28 showing moderate granulomatous infiltration (arrows), bar =200 μm.

**Figure 9:**
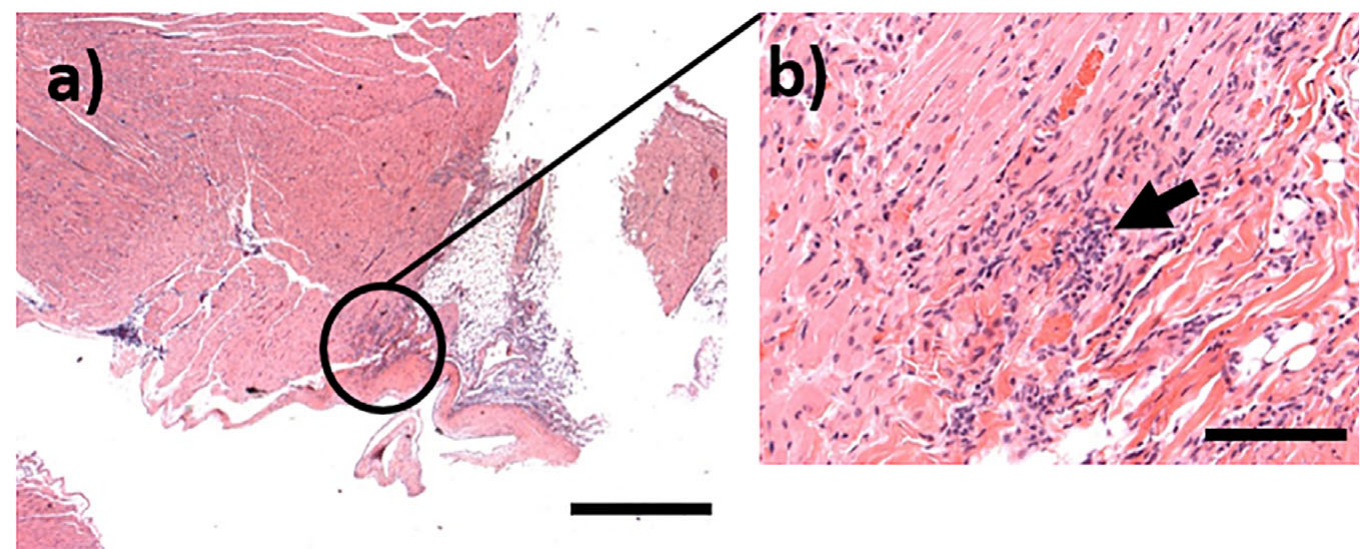
Representative H&E stained sections from marmosets challenged with *C. burnetii.* **(A)** Focal infiltration of the myocardium in an animal euthanasied at Day 14 post-challenge, bar =1mm; **(B)** High magnification image from **(A)** showing mixed inflammatory cells within the foci (arrow), bar =100μm.

Microscopic changes consistent with infection by *C. burnetii* were not observed in the tissues of any animals euthanized on day 3 p.c. By day 7 p.c., mild to moderate lesions, consistent with infection were present in the lungs of all animals (**Figure 8**). These comprised of a patchy to diffuse, inflammatory cell infiltrate, characterised mainly by neutrophils and macrophages, with scattered lymphocytes (pyogranulomatous alveolitis) (**Figures 8B**). These cells expanded the alveolar wall, and focally effaced and replaced normal parenchyma. Mild acute, focal bronchiolitis was observed in 50% of the animals. This was characterized by inflammatory cell infiltration composed mainly by degenerated and some viable neutrophils, with some macrophages, lymphocytes and plasma cells infiltrating the walls and lumen of bronchioles (**Figure 8C**). By day 14 p.c., mild to severe lesions were observed in the lungs of all four animals. The prominence of neutrophils in the inflammatory response had reduced, and patchy to diffuse, granulomatous alveolitis was observed, characterized by the presence of a majority of macrophages and lymphocytes. Minimal, acute bronchiolitis was seen in the lungs of one animal. Minimal to mild perivascular cuffing of inflammatory cells was observed in two animals (**Figure 8D**). Minimal to mild vasculitis was observed in the same animals. A focus of inflammatory cell infiltration, composed mainly by macrophages, lymphocytes and plasma cells was observed in the heart of one animal, infiltrating an atrial wall, and extending into the epicardium and adjacent fibro-vascular adipose tissue (**Figure 9**). Lesions consistent with infection by *C. burnetii* were observed in ten out of twelve animals euthanised at day 21 p.c. from the dose ranging study. The animals challenged with the lowest dose (<10 cfu) did not have any observable lesion in the lung. In the remaining animals, lesions comprised of multifocal to patchy infiltrations of macrophages and lymphocytes which expanded alveolar walls and spaces (granulomatous alveolitis), with no correlation to challenge dose (**Figure 8E**). In addition, lymphocytic, perivascular cuffing was also observed with mild to moderate severity. By day 28 p.c., chronic inflammatory changes were still observable in all animals.

## Discussion

The aim of this study was to develop a model of inhalational Q fever using the common marmoset. In humans, Q fever has a pleomorphic clinical presentation, ranging from an acute, febrile self-limiting disease to a chronic, sometimes fatal, disease with multiple organ involvement. Approximately 40% of patients are symptomatic, with disease severity and incubation period associated with infectious dose ( Benenson and Tigertt, 1956; Brooke et al., 2013; Heppell et al., 2017). In acute Q fever, febrile disease is common, with patients presenting with headaches, anorexia, atypical pneumonia or acute hepatitis, indicated by deranged liver enzymes activities such as increased aspartate transaminase (AST) and alanine transaminase (ALT) levels. Further, around 20% of patients will develop Q fever chronic fatigue syndrome, a debilitating sequelae resulting in long-term detrimental effects on daily function (Kampschreur et al., 2012).

The marmoset exhibited many features of human acute Q fever, presenting as a self-limiting, febrile disease. In humans, the duration length of fever is 5 to 14 days in untreated patients, compared with to 4 to 11 days in the marmoset (Derrick, 1973). During pyrexia in marmosets, weight loss was apparent although not as significant as observed in other animal models (Russell-Lodrigue et al., 2006; Norville et al., 2014), Weight loss in humans is reported in some cases (Schneeberger et al., 2014); in particular in association with endocarditis and other Q fever-associated complications (Maurin and Raoult, 1999). As Q fever progresses in humans, bacteria can be isolated from the blood and increasing antibody titres indicates seroconversion has occurred (Schneeberger et al., 2010). Similarly, in our study, one marmoset was bacteraemic by 3 p.c, and all animals were between day 7 and 14 p.c. No bacteria were observed in the blood after day 14 although viable bacteria were observed in the lungs of 75% of animals at day 21 and day 28 p.c.

Previously Q fever models have relied upon qPCR to determine bacterial load within tissues due to the difficulty in culturing viable *C. burnetii*. qPCR has the disadvantage of detecting DNA from both live and dead bacteria and therefore provides limited information on bacterial clearance as tissues can still appear colonised due to the persistence of DNA, but are clear of viable organisms. We have previously demonstrated in mice that using viable counts to monitor disease progression allows a more reliable representation of pathogenesis (Hartley et al., 2019). In addition, the mice were shown still to be colonised at day 22 p.c. It has also been reported that culturable bacteria were found in both the bones and the adipose tissue indicating that these tissues are potentially sources of long term colonization (Marmion et al., 2009; Bechah et al., 2014). Bacteria were cultured from these tissues in the marmoset model, but only at the peak of infection.

Q fever is diagnosed in humans using serology and increasingly PCR (Fournier and Raoult, 2003; Wielders et al., 2015; Bae et al., 2019). In this study, a human diagnostic test (NovaTech ELISA) was used in order to directly compare marmoset and human serological responses. Using the human diagnostic interpretation of these results (Phase II IgG antibody titres > Phase I IgG antibody titres), the data indicates the development of an acute infection in the marmoset. With time the decline in Phase II antibodies in favour of Phase I antibodies was expected, although this occurred faster in the marmosets than in humans (Wielders et al., 2015), possibly due to the higher infecting dose. Marmoset blood samples tested positive by PCR from day 7 to 28, the end of the study, comparable to other NHP studies and human results (Howe et al., 2009; Bae et al., 2019). The utility of a simple throat swab was demonstrated to have utility for diagnosis (positive in 50% of the animals) before seroconversion. No overt disease was observed in the two marmosets that received less than 10 cfu of *C. burnetii*, however one of the animals had *C. burnetii* specific antibodies detected in the plasma. Indeed, in humans Q fever is not always associated with disease with 60% of individuals being asymptomatic and disease severity and incubation period is dose-dependent (Brooke et al., 2013).

Liver dysfunction is associated with human Q fever, with elevated levels reported in 85% of cases (Fournier et al., 1998; Wielders et al., 2014). In the marmoset there was elevation of two liver enzymes, GGT and ALKP at the same time as fever and weight loss, indicating they may be a useful early indicator of disease. Contrastingly, the two other liver enzymes, AST and ALT, are elevated at the end of the study (Day 21). Elevation of these enzymes may persist for several months after acute disease.

Rarely (<5%) Q fever human patients will develop a chronic form of the disease, with symptoms ranging from fatal endocarditis to a debilitating chronic fatigue syndrome (Maurin and Raoult, 1999). This aspect of the disease was not explored in the marmoset. However, cellular infiltration was observed in the myocardium of one animal although further work is required to determine whether this was associated with bacterial entry.

The marmosets raised a robust immunological response initially involving neutrophils, but also circulating monocytes and lung macrophages. Significant levels of circulating INF-*γ* were produced coinciding with the peak infection, causing classical activation of the macrophages and bringing the infection under control (Dellacasagrande et al., 1999; Benoit et al., 2008). T cell activation was observed during the recovery stage of the disease, their activity has been shown to be critical in clearance in other animal models, (Andoh et al*.*, 2007). IFN-*γ* production in a re-call assay and the seroconversion of the animals suggests that these animals raised a protective response. Both a cell mediated T cell response and Coxiella specific antibodies are considered important indicators of successful vaccination in humans (Kersh et al., 2013; Schoffelen et al., 2013; Chen et al., 2019). As the immune response followed the expect pattern demonstrates that this is an appropriate animal model to assess protective measures.

Due to the non-lethal nature of the disease, suitable biomarkers of infection are critical to assess the efficacy of medical countermeasures. This would require a multifaceted approach looking at both generic and specific indicators of disease. In the marmoset model, protection could be assessed using a primary indicator of absence of fever in conjunction with secondary indicators of lack of weight loss, bacteraemia, CD64^+^ activated neutrophils, CD40^+^ positive macrophages and liver dysfunction. Some of this data can be collected without handling animals; fever can be monitored remotely using telemetry, as utilized in the current study, and weight assessed from animals trained to remain on balances for data collection. However, assessment of the other indicators, such as bacteraemia, would require handling and timely blood withdrawals during the febrile period.

The marmoset model recapitulates many aspects of human Q fever and therefore offers a scientifically valid alternative model to assess medical countermeasures. Previous studies have routinely used mice and guinea pigs to model Q fever and assess vaccines. Both species demonstrate limited features of human acute Q fever, such as anorexia, splenomegaly and evidence of pneumonia (Metters et al., 2019). These models certainly have a place in assessing vaccines and therapies. However, higher order animals such as NHPs are typically required as preclinical models for licensure purposes. Therefore, well characterized NHP models of Q fever are necessary. Previous work used old world primates such as the cynomologus and rhesus macaques and, similar to the marmoset model, exhibited bacteraemia and fever (Waag et al., 1999). However, these more traditional NHP models are in high demand and are expensive and challenging to work with under high containment conditions (Patterson and Carrion, 2005). The marmoset is a New World monkey and while it is a lower order primate, it still has a high immunological similarity to humans (Nelson and Loveday, 2014). The use of this small primate to study infectious disease is on the rise with more laboratories embracing this species (Carrion and Patterson, 2012). In the case of Q fever, the marmoset model exhibits similar disease but has the advantage of being a lower order animals and, due to its small size is easy to handle within high containment.

In these studies, marmosets have been shown to be susceptible to infection with *C. burnetii*, in a dose-dependent manner. These are the first studies showing the spread and clearance of viable bacteria in an NHP model. As infection in the marmoset is non-lethal, key biomarkers of disease have been identified to measure disease progression. These biomarkers can be used in proof-of-concept studies determine whether the marmoset are a suitable model to assess medical countermeasures.

## Data Availability Statement

The original contributions presented in the study are included in the article/supplementary materials. Further inquiries can be directed to the corresponding author.

## Ethics Statement

The animal study was reviewed and approved by Home Office Project licence P18072309.

## Author Contributions

MN designed, performed, and analyzed the study as well as wrote the manuscript. LH performed and analyzed the histology work. FS designed, analyzed, and reviewed the histology work. TA technically reviewed the work. All authors contributed to the article and approved the submitted version.

## Conflict of Interest

The authors declare that the research was conducted in the absence of any commercial or financial relationships that could be construed as a potential conflict of interest.
